# Supplementary material for: Clinical profile of HIV-infected adults receiving a holistic approach of care model in Nakawa, Kampala District
Source: PLoS One. 2022 Jul 26;17(7):e0272039. doi: 10.1371/journal.pone.0272039 (PMC9321371; doi:10.1371/journal.pone.0272039)
Supplement: Attachment — Submitted filename: Response to Reviewers.docx [file pone.0272039.s002.docx]

**Contact us at:**

Reach Out Mbuya Community Health Initiative

P. O. Box 7303 Kampala Uganda

Tel: +256 414 222 630 / + 256 312 165 250

Email: [reachout@reachoutmbuya.org](mailto:reachout@reachoutmbuya.org)

Website: www.reachoutmbuya.org

**A Holistic model of care**

Medical Care

Social Support

Community Network

**Date 15/03/2022**

Dear Editor

**RE:** **REVISED SUBMISSIONS; PONE-D-21-11284 “CLINICAL PROFILE OF HIV-INFECTED ADULTS RECEIVING A HOLISTIC APPROACH OF CARE MODEL IN NAKAWA, KAMPALA DISTRICT”**

Thank you for taking the time to review the Manuscript that was submitted and for the comments/ suggestions proposed. Below are the responses to the comments

**Comment:** Is the manuscript technically sound, and do the data support the conclusions?

The manuscript must describe a technically sound piece of scientific research with data that supports the conclusions. Experiments must have been conducted rigorously, with appropriate controls, replication, and sample sizes. The conclusions must be drawn appropriately based on the data presented.

Reviewer #1: Partly

Reviewer #2: Partly

**Response: N/A**

**Comment:** Has the statistical analysis been performed appropriately and rigorously?

Reviewer #1: Yes

Reviewer #2: Yes

**Response: N/A**

**Comment:** Have the authors made all data underlying the findings in their manuscript fully available?

The [PLOS Data policy](http://www.plosone.org/static/policies.action#sharing) requires authors to make all data underlying the findings described in their manuscript fully available without restriction, with rare exception (please refer to the Data Availability Statement in the manuscript PDF file). The data should be provided as part of the manuscript or its supporting information, or deposited to a public repository. For example, in addition to summary statistics, the data points behind means, medians and variance measures should be available. If there are restrictions on publicly sharing data—e.g. participant privacy or use of data from a third party—those must be specified.

Reviewer #1: Yes

Reviewer #2: Yes

**Response: N/A**

**Comment:** Is the manuscript presented in an intelligible fashion and written in standard English?

PLOS ONE does not copyedit accepted manuscripts, so the language in submitted articles must be clear, correct, and unambiguous. Any typographical or grammatical errors should be corrected at revision, so please note any specific errors here.

Reviewer #1: Yes

Reviewer #2: No

**Response: N/A**

Review Comments to the Author

**Comment:** Line 46: Could the author clarify what the 2020-mile stone is about?

**Response:** The statement was out of recent context; the line has been revised.

**Comment:** Line 58 and 63 appeared repetitive, could the author revise this without the repetitions?

**Response:** Both lines have been revised to give a clear meaning.

**Comment:** The sentence towards the end of line 73-75 seems incomplete although it read like the aim/objective of the study, therefore author should review this sentence as appropriate.

**Response:** The sentence has been revised

**Comment:** Line 80-81 Could the author describe what a holistic approach of care model is? What specifically were the patients receiving or what are the care components?

**Response:** The holistic approach has been described in the introduction section. Line 66 -74

**Comment:** Line 104-105 Could the author state the ethics approval reference numbers for the 2 ethics bodies?

**Response:** All ethical approval reference numbers have been updated

**Comment:** Line 158 could the author clarify if they are writing viral load or viral road?

**Response:** Typo error corrected

**Comment:** Line 162 Could the author stick to either 'percent' or '%' for consistency

**Response:** The manuscript is revised to %

**Comment:** Line 167-169 Could the author revise this statement about 'viral load non-suppression' its a bit confusing to say 'reduced the likelihood of viral load non-suppression by 65%'. If the author is reporting a reduction in viral load suppression by 65%, then state that, and avoid the term 'non-suppression'. This has been mentioned a few times throughout the manuscript, please review and revise.

**Response:** The manuscript analysis has been revised to reflect viral load suppression

**Comment:** Line 180-181 Could the author define the abbreviations used

**Response:** The abbreviations used have been defined.

**Comment:** Line 183-186 Could the author revise the strengths of this study, as this cannot only focus on using a ‘well-trained professional’ to collect data.

**Response:** Study strengths have been revised.

**Comment:** Line 194-196 This final statement is not quite clear...what is the relationship between age, adherence and the use of holistic approaches?

**Response: N/A**

**Reviewer #2: The authors reported the clinical profiles of PLHIV receiving holistic care in Nakawa. There are some comments and questions below**
Abstract
**Comment:** The reference citation is not needed in the abstract

**Response:** The abstract has been revived

**Comment:** The numbers should not be used for the beginning of the sentences

**Response:** The manuscript has been revised

**Comment:** The authors said: 84.5% had VL detectable, and 95.3% had UD VL. Which number is corrected?

**Response:** The statement has been revised

**Comment:** The authors concluded that holistic care had good effects. However, there is no data on comparing "not holistic care."
Text

**Response:** The reviewer is right, there was no comparator. This was basically a cross sectional study. Generally cross-sectional studies have that weakness in common

**Comment:** The reference citation is not needed in the abstract. Thus, it should start with 1 in the main text section

Response: All references have been revised and updated

**Comment:** UNAIDS data is needed to be updated

**Response:** Reference has been updated

**Comment:** What is the definition of holistic care/system? Please explain in detail and what is the difference from the standard/routine care in the method section

**Response:** The holistic approach has been described in the introduction section. Line 66 -74

**Comment:** The study should be a retrospective cohort study rather than a cross-sectional study

**Response:** With several technical consultations, we believe that the study is retrospective

cross-sectional study rather than a a retrospective cohort.

**Comment:** Why do the authors use stratified random sampling? Does this mean that all areas there use a holistic system?

**Response:** Yes, all study facilities use a holistic approach

**Comment:** If possible, systemic randomized should not be used due to the bias. Please explain why using this sampling method

**Response:** We acknowledge the weaknesses of systemic randomized however will also weighed the strength and simplicity it could introduce to the study such as eliminating the phenomenon of clustered selection

**Comment:** What is the statistical program used in this study?

**Response:** STATA version 13. 0 mentioned in line 25

**Comment:** Tables should be moved to the back, and more texts are needed to describe the details of the results

**Response:** This has been done

**Comment:** The abbreviation should be stated under each table

**Response:** This has been done

**Comment:** Table 4, what is the definition of good adherence?

**Response:** Defined in line 142

**Comment:** Table 3 and 4, for continuous variables, please try to analyze as a continuous variable and compare the results

**Response:** Re-analysis done, Age as a continuous variable destabilized the model fit and other variables are binary or ordinal by nature

**Comment:** Table 4, why use the outcome of VL non-suppression. But the authors discuss VL suppression.

**Response:** Re-analysis done, VL suppression is currently the outcome

**Comment:** Please check references: order and format (e.g., authors list and name of the journal)
Others

**Response:** References have been revised and reorganized.

**Comment:** English language is needed to be correct

**Response:** English language has been corrected

**Comment:** More details in main methods, results, and discuss section are needed

**Response:** All sections have been updated.

Thank you so much for your guidance on this study and I look forward to hearing from you.

Yours,

Sendaula Emmanuel
